# Integrated analysis of SR-like protein kinases Sky1 and Sky2 links signaling networks with transcriptional regulation in *Candida albicans*

**DOI:** 10.3389/fcimb.2023.1108235

**Published:** 2023-04-03

**Authors:** Christian H. Luther, Philipp Brandt, Slavena Vylkova, Thomas Dandekar, Tobias Müller, Marcus Dittrich

**Affiliations:** ^1^ University of Würzburg, Department of Bioinformatics, Biocenter/Am Hubland 97074, Würzburg, Germany; ^2^ Septomics Research Center, Friedrich Schiller University and Leibniz Institute for Natural Product Research and Infection Biology – Hans Knöll Institute, Jena, Germany; ^3^ University of Würzburg, Institut of Human Genetics, Biocenter/Am Hubland 97074, Würzburg, Germany

**Keywords:** sky kinases, kinase signaling, network analysis, *Candida albicans*, transcriptome, transcriptional regulation, phosphoproteome

## Abstract

Fungal infections are a major global health burden where *Candida albicans* is among the most common fungal pathogen in humans and is a common cause of invasive candidiasis. Fungal phenotypes, such as those related to morphology, proliferation and virulence are mainly driven by gene expression, which is primarily regulated by kinase signaling cascades. Serine-arginine (SR) protein kinases are highly conserved among eukaryotes and are involved in major transcriptional processes in human and *S. cerevisiae*. *Candida albicans* harbors two SR protein kinases, while Sky2 is important for metabolic adaptation, Sky1 has similar functions as in *S. cerevisiae*. To investigate the role of these SR kinases for the regulation of transcriptional responses in *C. albicans*, we performed RNA sequencing of *sky1*Δ and *sky2*Δ and integrated a comprehensive phosphoproteome dataset of these mutants. Using a Systems Biology approach, we study transcriptional regulation in the context of kinase signaling networks. Transcriptomic enrichment analysis indicates that pathways involved in the regulation of gene expression are downregulated and mitochondrial processes are upregulated in *sky1*Δ. In *sky2*Δ, primarily metabolic processes are affected, especially for arginine, and we observed that arginine-induced hyphae formation is impaired in *sky2*Δ. In addition, our analysis identifies several transcription factors as potential drivers of the transcriptional response. Among these, a core set is shared between both kinase knockouts, but it appears to regulate different subsets of target genes. To elucidate these diverse regulatory patterns, we created network modules by integrating the data of site-specific protein phosphorylation and gene expression with kinase-substrate predictions and protein-protein interactions. These integrated signaling modules reveal shared parts but also highlight specific patterns characteristic for each kinase. Interestingly, the modules contain many proteins involved in fungal morphogenesis and stress response. Accordingly, experimental phenotyping shows a higher resistance to Hygromycin B for *sky1*Δ. Thus, our study demonstrates that a combination of computational approaches with integration of experimental data can offer a new systems biological perspective on the complex network of signaling and transcription. With that, the investigation of the interface between signaling and transcriptional regulation in *C. albicans* provides a deeper insight into how cellular mechanisms can shape the phenotype.

## 1 Introduction

Fungal infections constitute a major health burden worldwide with more than 150 million people suffering from serious fungal diseases. *Candida albicans* is one of the most common fungal pathogens of humans and is frequently found on the mucosal surfaces of the body. Invasive candidiasis poses a major threat with more than ~700,000 cases worldwide (Bongomin et al., 2017) and *C. albicans* is the most common cause of invasive candidiasis (Pfaller and Diekema, 2007) with very high mortality rates of 40 to 60% (Hsueh et al., 2009; Bassetti et al., 2019).

Gene expression plays a major role for the regulation of cellular processes shaping the phenotype by altering the morphology, growth and proliferation of the fungal cells which can lead to increased or decreased virulence or drug resistance of a fungal pathogen. Thus, detailed knowledge about the regulation of transcriptional processes is the key to understand how cellular functions shape the phenotype. Gene expression is one of the major downstream effectors of many signaling cascades, most of which are primarily built around central kinases which trigger cascading protein phosphorylations. These signaling cascades typically form complex networks, rather than linear pathways. Thus, to gain deeper insight into cellular functions, integrated methods of network analysis and large amount of data are required. This relates not only to the measurement of proteins, phosphorylation and transcripts levels, but also to information on how the components interact with each other.

Nowadays, high throughput technologies are becoming more available and affordable. In particular, mass spectrometry and Next Generation Sequencing (NGS) provide large scale profiles of the cellular transcriptome, proteome and phosphoproteome. Phosphoproteomics enables the characterization of proteins that are post-translationally modified by addition of a phosphate group and phosphopeptide enrichment methods can identify several thousands of phosphoproteins in one experiment (Zhou et al., 2010). To date, tens to hundreds of thousands of phosphosites of serine, threonine, or tyrosine residues have been identified in many organisms, including human (Ochoa et al., 2020), mouse (Huttlin et al., 2010; Qi et al., 2014) and yeast (Vlastaridis et al., 2017; Lanz et al., 2021). Several studies in fungi (Ramsubramaniam et al., 2014; Ren et al., 2016; Mattos et al., 2020) have exploited these technical possibilities to investigate the evolution of kinases and identify new substrates in different fungal species. most efforts focus on the study of established model organisms, while the characterization of the phosphoproteome of many fungi is still at an early stage. However, most efforts, focus on the study of established model organisms, while in many fungi, characterization of the phosphoproteome is still at an early stage. Nevertheless, it remains a major challenge to understand how this data translates into knowledge about the function of the cellular system. Cellular functions that shape fungal phenotypes are the product of a complex interplay between genes and proteins. Thus, to understand regulatory processes different cellular levels such as the phosphoproteome (delivering a view on signaling) and the transcriptome (capturing the effect of downstream gene expression) need to be measured in the same strain under the same conditions. Most important, but challenging, is the integration and subsequent analysis of the data in a common framework.

Serine-arginine (SR) protein kinases are highly conserved in eukaryotes (Giannakouros et al., 2011). They are characterized by phosphorylating SR proteins, which are enriched in serine/arginine recognition motifs at their N-terminus (Ghosh and Adams, 2011). In humans, SR proteins regulate major RNA related processes like mRNA export from the nucleus and both alternative and constitutive splicing (Zhou and Fu, 2013). In contrast, splicing plays only a minor role in the yeast *S. cerevisiae* but has been described to play a regulatory role during stress response in *C. albicans* (Sieber et al., 2018). However, it has been shown that the only SR kinase in *S. cerevisiae*, Sky1, phosphorylates the SR-like protein Npl3, which regulates pre-mRNA splicing and mRNA export from the nucleus (Moehle et al., 2012). In addition, Sky1 is important for polyamine transport and maintenance of ion homeostasis (Erez and Kahana, 2001; Forment et al., 2002). Recently, the function of SR kinases in *C. albicans* was described for the first time in human pathogenic fungi (Brandt et al., 2022). In this study, we performed a phylogenetic analysis which revealed that most common pathogenic Candida species possess two Sky kinases (Sky1 and Sky2), with the exception of *C. glabrata*, which contains only one SR protein kinase (Sky1 group)(Brandt et al., 2022). Furthermore, Sky1 and Sky2 have large sequence differences, indicating that these kinases have different functions in *C. albicans*. It has been shown that the SR-like protein kinase Sky1 in *C. albicans* appears to have a similar function as in *S. cerevisiae* (Brandt et al., 2022). In contrast, the SR-like protein kinase Sky2 is important for the metabolism of dipeptides and thus for the metabolic adaptation of the fungus (Brandt et al., 2022). Identifying the role of SR kinases in the human fungal pathogen *Candida albicans* could provide new insights into its virulence mechanisms.

Here, we set out to investigate the effects of SR-like kinase on transcriptional regulation in *C. albicans* and established integrated modules of Sky1 and Sky2 kinase signaling, focusing on downstream effects on gene expression. Therefore, we performed RNA-Seq to generate transcriptional profiles of *sky1*Δ and *sky2*Δ mutant strains and integrated data from a phosphoproteome study of these mutants, both comparing mutants with wild type (WT) controls. Subsequently, we performed pathway and transcription factor enrichment analysis to identify key components of transcriptional regulation. Next, we integrated computational predictions of kinases for the phosphoproteome data and further assembled information on protein-protein interactions to provide linking information between the signaling proteins and the transcription factors. Combining all data into a joint network we derived signaling modules for each mutant strain, which allow the analysis of transcriptomic and phosphoproteomic profiles within in this network context. This provides a detailed view on the interface between cellular signaling cascades and transcriptional regulation which offers the opportunity to investigate not only direct but also indirect effects of SR-like kinases in *C. albicans*. With that, we analyze specific and common functions of the two Sky kinases in a comparative manner, with focus on their influence on transcriptional regulation. We derive and discuss potential mechanisms and functional implications of various regulatory interactions. Here, we demonstrate how the combination of experimental data with network information can offer a broader perspective on *C. albicans* Sky kinases and can help to generate novel hypotheses about how signaling and transcription can shape the fungal phenotype.

## 2 Material und methods

### 2.1 *Candida albicans* strains used in this study

The *C. albicans* strains used in this study are listed in **Supplementary Table 1**. All strains were stored as frozen stocks containing 20% glycerol at -80°C and sub-cultured on YPD agar plates (1% yeast extract, 2% peptone, 2% glucose, 2% agar) at 30°C for 2 days. Strains were routinely grown in YPD liquid medium at 30°C overnight with shaking at 180 rpm.

### 2.2 Spot dilution assays

Sensitivity of the strains to the antibiotic Hygromycin B was tested by spot dilution assays on YPD agar plates. YPD overnight cultures of the wild type and mutant strains were centrifuged (4,000 × g, 5 min) and washed with dH_2_O. Strains were adjusted to an optical density of 600 nm (OD_600_) of 1.0. 5 µl of each 10-fold serial dilutions were spotted onto the YPD plates without or with Hygromycin B (250 µg/ml or 300 µg/ml) and incubated for 3 days at 37°C.

### 2.3 Hyphae formation assay

YPD overnight cultures of the wild type and mutant strains were centrifuged (4,000 × g, 5 min), washed with dH_2_O, adjusted to an OD_600_ of 0.5 and 5 µl of each cell suspension was spotted on solid arginine medium. The plates were incubated for 3 days at 37°C. Two different types of arginine media were used: 0.17% (w/v) yeast nitrogen base without ammonium sulfate and amino acids, 0.5% (w/v) ammonium sulfate, 1% (w/v) arginine (pH 6), and 0.17% (w/v) yeast nitrogen base without ammonium sulfate and amino acids, 10 mM Arginine (w/v), 0.2% glucose (w/v; pH 6).

### 2.4 Transcriptomic profiling

YPD (1% yeast extract, 2% peptone, 2% glucose) overnight cultures of the *C. albicans* wild type as well as the *sky1*Δ and *sky2*Δ mutant strains were diluted in 50 ml fresh YPD medium with a starting OD_600_ of 0.3 and grown for 3 h at 37°C to logarithmic growth phase. Subsequently the strains were centrifuged (4000 × g, 5 min), washed with dH_2_O and were adjusted to an OD_600_ of 0.2 in 30 ml YPD medium and grown at 37°C for 4 h. Cells were collected by centrifugation and the cell pellets were shock frozen with liquid nitrogen. The cell pellets were resuspended in 400 µl AE buffer (50 mM sodium acetate, 10 mM EDTA) and transferred to screw cab tubes followed by adding 40 µl 10% SDS solution, 500 µl glass beads and 440 µl Phenol-Chloroform-Isoamylalkohol 125:24:1 (Ambion, acidic pH). Cells were mechanically disrupted on a FastPrep-24 cell-homogenizer (MP Biomedicals, Santa Ana, USA) with two 40 s runs at 6.0 m/s and with 5 min on ice incubation between each run. Samples were centrifuged at 12,000 g for 2 min and the upper phase was transferred into a new reaction tube and mixed with 1/10 volume 3 M sodium acetate (pH 5.3) and one volume 2-propanol. To precipitate the RNA, the samples were incubated for 30 min at -20°C and subsequently centrifuged for 10 min at 12,000 g. The pellets were washed twice with 100 µl 70% ethanol and resuspended in 100 µl RNase-free water. RNA quality and quantity were determined using the RNA Nano 6000 Assay Kit of the Bioanalyzer 2100 system (Agilent Technologies, CA, USA) and the NanoPhotometer^®^ spectrophotometer (IMPLEN, CA, USA), respectively. RNA sequencing was performed by Novogene (Cambridge, United Kingdom) using the Illumina NovaSeq 6000 Sequencing System. The mRNA was enriched by a polyA capture followed by reverse transcription of cDNA to prepare an RNA library. Illumina PE150 technology was used for 150 bp paired-end sequencing.

### 2.5 Transcriptomic analysis

After quality control of the libraries, all reads have been mapped against the *C. albicans* genome (Haplotype A chromosomes only, A22-s07-m01-r142 version, obtained from the Candida Genome Database (Skrzypek et al., 2017) with STAR (version 2.7.5b) (Dobin et al., 2013). Subsequently, aligned reads have been counted in a gene-wise manner using ‘featurecounts’ from the ‘Rsubread’ (version 2.0.1) package (Liao et al., 2014). Differential expression analysis has been performed based on generalized linear models comparing each mutant group with the control group as implemented in DESeq2 (version 1.26) (Love et al., 2014). Genes with an adjusted P-value < 0.05 (Benjamini and Hochberg, 1995) have been considered as significant and have been categorized as ‘low’ (absolute log_2_FC < 0.5) or ‘moderate to high’ (absolute log_2_FC ≥ 0.5) for further analyses. All transcriptomic analyses have been performed with the statistical software package R (version 3.6.3). Transcription factor enrichment was performed *via* the PathoYeastract+ website (Monteiro et al., 2020). Pathway analysis of at least moderately regulated genes were performed *via* the R-Package of g:Profiler (Kolberg et al., 2020).

### 2.6 Biological annotation of interactors

A comprehensive repertoire of the *C. albicans* kinome has been compiled from literature (Lee et al., 2016) and was complemented by an automatic classification of kinases into superfamily, family and subfamily using Kinannote 1.0 (Goldberg et al., 2013). Phenotypic profiles for 143 C*. albicans* transcription factors were obtained from a published high-throughput knockout experiment (Homann et al., 2009). Furthermore, experimentally validated direct and indirect transcription regulatory associations of 127 transcription factors were extracted from PathoYeastract+ (Monteiro et al., 2020). Transcription regulatory genes from both sources were combined to obtain a comprehensive list of *C. albicans* transcription factors. Structure of the arginine pathway was obtained from KEGG (Okuda et al., 2008).

### 2.7 Construction of an integrated *C. albicans* signaling network for *sky1*Δ and *sky2*Δ mutants

As basis for the construction of *sky1*Δ and *sky2*Δ modules, integrated *C. albicans* signaling networks were created combining kinase-substrate interaction (KSI) and protein-protein interaction data. Significant differentially phosphorylated kinases and transcription factors (absolute log_2_FC ≥ 1 and adjusted P-value < 0.05 obtained from phosphoproteomic datasets of *sky1*Δ and *sky2*Δ in YPD ((Brandt et al., 2022); PXD027612; **Supplementary Figure 1A**) were analyzed with the stand-alone batch predictor package of the Group-based Prediction System (GPS) (Wang et al., 2020). The high threshold option and the UniProt *C. albicans* reference proteome (UP000000559; downloaded August 2019) were used to predict site-specific phosphorylation for 617 different classifiers on the level of group, family and subfamily. To obtain kinase-specific information, the proteins of the *C. albicans* kinome were mapped to the corresponding classifiers, matching the kinase group, family and subfamily provided by the GPS prediction with that classified by Kinannote 1.0 (Goldberg et al., 2013). To remove low confidence predictions, only interaction with a GPS5 score greater than 80 were further used. As no information on experimentally validated KSI for *C. albicans* is available from databases so far, kinase-substrate interactions of *S. cerevisiae* obtained from major publicly available databases (dbPAF (Ullah et al., 2016), HPRD (Keshava Prasad et al., 2009), Phospho.Elm (Dinkel et al., 2011), PhosphoGRID (Oughtred et al., 2021), PhosphoSitePlus (Hornbeck et al., 2015), YeastKinome (Breitkreutz et al., 2010)) were transferred *via* the well-established interolog method as described in Remmele et al. (2015). Briefly, this method transfers interactions from template species *S. cerevisiae* to a target species (*C. albicans*) by linking two proteins in the target species by an interaction (A’ and B’) if the orthologous proteins in the template species are known to interact (A-B; **Supplementary Figure 1B**). For this, the orthology relation of proteins were extracted from the Ensembl Compara database (version 96) (Herrero et al., 2016) from the public ensemble MySql-Server (mysql-eg-publicsql.ebi.ac.uk:4157). Then the multiple sequence alignments of the respective Compara families were used to transfer the phosphorylation sites to orthologous proteins and sites in *C. albicans*. To derive a comprehensive protein-protein interaction network of *C. albicans*, experimentally validated protein-protein interactions of *C. albicans* were obtained from the 14 active partners of the International Molecular Exchange (IMEx) (Orchard et al., 2012) *via* PSICQUIC (searched on January, 2020) (Aranda et al., 2011). Additionally, all interactions were tested for support of domain-domain interactions. For that, the domains of the network interactors (A’, B’) were obtained from the UniProt website in January 2021 (UniProt, 2022). The information of known domain–domain interactions were downloaded from the database of three-interacting domains (3did) (Mosca et al., 2014) in January 2021 (**Supplementary Figure 1B**). This network was then filtered for interactions of differentially phosphorylated or expressed interactors which interact with kinases or transcription factors. From this filtered network we extracted the largest connected subnetwork for *sky1*Δ and *sky2*Δ (**Supplementary Figure 1C**).

## 3 Results and discussion

### 3.1 Transcriptomic profiling of *sky1*Δ and *sky2*Δ mutants reveals distinct transcriptional responses for each kinase

**Figure 1:**
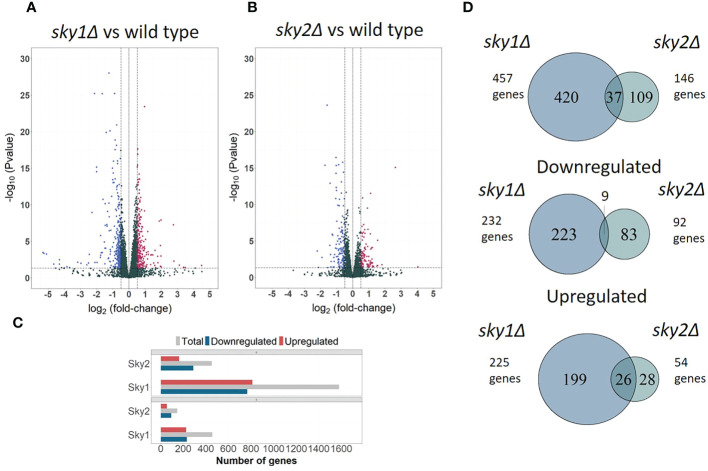
Transcriptional profiles of *sky1*Δ and *sky2*Δ compared to wild type reveals distinct responses for each kinase mutant. Volcano plots comparing transcriptomic abundance log_2_FC changes (X-axis) and the adjusted P-values (Y-axis) for **(A)** s*ky1*Δ and **(B)** *sky2*Δ versus wild type. RNA-Sequencing was performed after 4 hours growth of wild type, *sky*1Δ and *sky2*Δ in YPD medium. **(C)** Bar plot showing total number of differentially expressed (upper panel) and moderately to highly regulated genes (lower panel). Grouped bars represent upregulated (red), downregulated (blue) and total number of genes (grey) for *sky1*Δ and *sky2*Δ. **(D)** Venn diagrams of the total, downregulated and upregulated at least moderate differentially expressed genes (log_2_FC ≥0.5 and adjusted P-value < 0.05) of *sky1*Δ and *sky2*Δ.

To investigate the *SKY1* and *SKY2* knockout effects on gene regulation, we performed a genome wide transcription profiling of *sky1*Δ and *sky2*Δ mutant strains in comparison to the SC5314 wild type (WT) control (**Figures 1A, B**). The resulting expression profiles covered almost the entire *C. albicans* transcriptome with 99.2% (6,163/6,213) genes measured in at least one sample. Differential expression analysis yielded a total of 1,580 (25.4% of the entire transcriptome) differentially expressed genes (adjusted P-value < 0.05) for *sky1*Δ and 452 (7.3% of the entire transcriptome) differentially expressed genes for *sky2*Δ (adjusted P-value < 0.05). The analysis shows a largely symmetric effect on up- and downregulation for *sky1*Δ (51.4% up and 48.5% down) whereas in *sky2*Δ the majority of genes (63.9%) were downregulated (**Figures 1A–C**). Focusing on the set of genes with moderate to high expression changes (absolute log_2_FC ≥ 0.5 and adjusted P-Value < 0.05) the symmetric and asymmetric response patterns remain similar (**Figures 1C**). The relatively small number of differentially regulated genes shared between both strains points towards largely distinct effects of the kinases on transcriptional regulation (**Figure 1D**). The analysis of the directional effect of gene regulation reveals that most of the genes that are differentially expressed in both knockout strains, are regulated in the same direction, (**Figure 1D**) suggesting that Sky1 and Sky2 play a similar role in the regulation of these genes. Interestingly, we observed no transcriptional change of the other, intact, Sky kinase gene in both deletion strains compared to the wild type strain (expression of Sky2 in the *sky1*Δ mutant: log_2_FC = 0.001; adjusted P-value = 0.99 and expression of Sky1 in the *sky2*Δ strain: log_2_FC = 0.06; adjusted P-value = 0.74). This suggests that the deletion of one *C. albicans* Sky kinase does not lead to a compensatory transcriptional activation of the other Sky kinase gene.

The major key players in signaling and transcriptional regulation are kinases and transcription factors. In total, we found 71/282 (25.2%) transcription factors in *sky1*Δ to be differentially expressed (19 upregulated; 51 downregulated) whereas in *sky2*Δ 14 (5.0% of 282) transcription factors were differentially regulated (nine upregulated; five downregulated). Similar to the small overlap of all differentially regulated genes, only five transcription factors (*EFH1*, *FLO8*, *SWC4*, *TUP1*, *ZSF1*) were differentially expressed by both *sky*Δ mutant strains, among which only *EFH1* show an expression log_2_FC change greater than 0.5 in both mutant strains (*sky1*Δ: 1.04; *sky2*Δ: 1.1). Since phosphorylations play a major role in the direct and indirect regulation of transcription factors we searched for differentially regulated kinases in *sky1*Δ and *sky2*Δ. Out of the 115 kinases in *C. albicans* were 29 kinases (25.2%) in *sky1*Δ and six kinases (5.2%) in *sky2*Δ differentially regulated compared to the wild type. Only two kinases (*NIK1*, *HRR25*) were differentially expressed in both knockout strains albeit only with a low expression change. This indicates, that both Sky kinases have only small similarities on transcriptional level regarding regulated kinases and transcription factors.

### 3.2 Pathway analyses indicate conserved functions for Sky1 whereas Sky2 seems to regulate primarily metabolic processes

**Figure 2:**
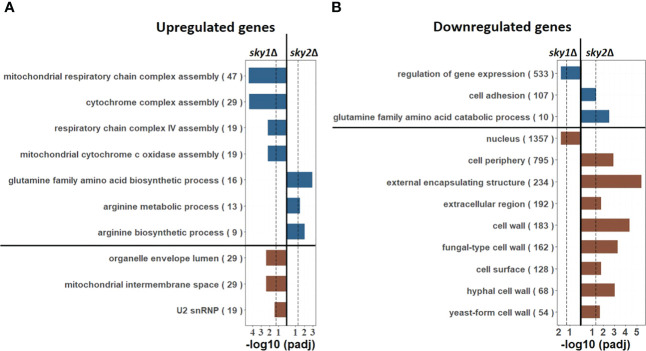
GO enrichment analysis reveals nuclear, mRNA processing and mitochondrial pathways for *sky1*Δ and glutamine and arginine metabolism pathways for *sky2*Δ. Bar plots show enriched biological processes (upper part; blue) and cellular components (lower part, brown) for **(A)** upregulated (moderate to high) as well as in **(B)** downregulated (moderate to high) genes. The X-axis represents the -log10 (adjusted P-value of enrichment) with the left side representing *sky1*Δ and the right side representing *sky2*Δ. For each GO category the total number of genes annotated in *C albicans* with this term is given in parentheses.

To functionally characterize the genome-wide transcriptional responses we performed pathway enrichment analyses based on the Gene Ontology (GO) categories, focusing again on genes with moderate to high effects. For *sky1*Δ this revealed an enrichment of mitochondrial respiratory associated categories like ‘mitochondrial respiratory chain complex assembly’, ‘mitochondrial intermembrane space’ and ‘organelle envelope lumen’ as well as RNA processing (‘U2 snRNP’) for upregulated genes (**Figure 2A**) and an enrichment of ‘regulation of gene expression’ and ‘nucleus localization’ for downregulated genes (**Figure 2B**). These processes are consistent with reported functions of Sky1 in *S. cerevisiae* where this kinase has been described to regulate various steps in RNA maturation and transport (Gilbert et al., 2001). In addition, a recent study has shown that the deletion of Sky1 in *S. cerevisiae* leads to mitochondrial defects (Hutchinson et al., 2022). In this light the significant upregulation of mitochondrial respiratory associated genes observed here indicates a similar mitochondrial involvement of Sky1 in *C. albicans*.

In contrast to that, we found an enrichment of metabolic and biosynthetic processes for arginine and glutamine for sky2Δ (**Figure 2**). For downregulated genes this relates primarily to catabolic processes (**Figure 2B**), whereas for upregulated genes primarily biosynthetic processes are enriched (**Figure 2A**) pointing towards a role of Sky2 in amino acid metabolism. Furthermore, the set of downregulated genes is primarily associated with the cell periphery (e.g. ‘hyphal cell wall’, ‘extracellular region’, ‘cell surface’, ‘cell periphery’). These results indicate that both kinases have different cellular functions in *C. albicans*. Similar to *S. cerevisiae*, Sky1 seems to be, mainly involved in mRNA processing as well as nuclear and mitochondrial related processes, whereas Sky2 seems plays a distinct role in the cell periphery and is involved in the transcriptional regulation of glutamine and arginine metabolism.

### 3.3 Sky2 influences arginine metabolic processes and arginine-induced hyphae formation

**Figure 3:**
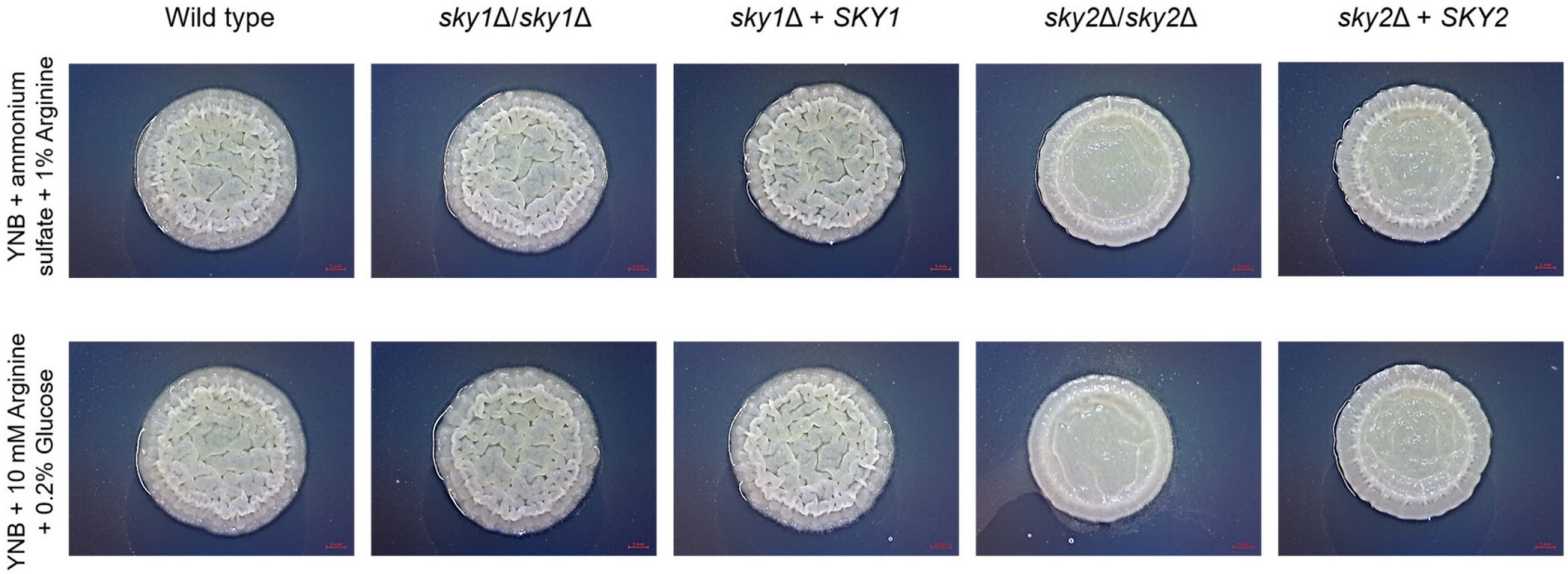
Arginine-induced wrinkle colony forming is impaired in the *sky2*Δ mutant. *C. albicans* strains were tested for arginine-induced filamentation on solid medium (0.17% YNB, 0.5% ammonium sulfate, 1% arginine [pH 6] and 0.17% YNB, 10 mM arginine, 0.2% glucose [pH 6]). The experiment was performed in duplicates and representative images were taken after 3 days of incubation at 37°C. The *SKY2* complemented strain partially rescued the *sky2*Δ mutant phenotype. This was also shown in a previous study [(Brandt et al., 2022) for detailed data see Supplementary Figure 3 there].

To further explore the role of Sky2 in amino acid sufficiency we focused on the differentially regulated genes in the glutamine and arginine metabolism pathways as provided by KEGG (Okuda et al., 2008). Interestingly, both *sky*Δ mutant strains showed a significant upregulation of the NADPH-dependent glutamate dehydrogenase (*GDH3*) which catalyzes the conversion of α-ketoglutarate and ammonium to glutamate. However, *sky1*Δ and *sky2*Δ mutants show different regulation of glutamate utilizing pathways like the biosynthetic and catabolic arginine pathway. So, *sky2*Δ upregulates most genes responsible for the conversion of glutamate to ornithine and further Arg4 which is the final step in the arginine biosynthesis pathway. This enzyme catalyzes the reversible breakdown of argininosuccinate to arginine and fumarate. Additionally, large parts of the arginine catabolic pathway with the initial conversion of arginine (Car1) to ornithine and further to proline, to glutamate (Put1, Put2) and to α-ketoglutarate as well as ammonium (Gdh2) are downregulated in *sky2*Δ. Since in this arginine catabolic pathway glutamate is used to fuel the TCA cycle (Silao et al., 2019), the *sky2*Δ strain seems to allocate glutamate for the generation of ornithine and arginine and instead of NADPH and ADP production. Sky1 has also been described in *S. cerevisiae* to be involved in the transport of polyamines and the maintenance of ion homeostasis (Erez and Kahana, 2001; Forment et al., 2002). Since ornithine is a precursor molecule for the polyamine production this could connect this intermediate of the arginine pathway with the known involvement of Sky1 orthologs in regulation of mRNA and polyamine transport. It has been shown that arginine and proline induce hyphae formation in *C. albicans* (Ghosh et al., 2009; Silao et al., 2019). As YPD contains arginine, we tested whether the *sky2*Δ mutant can filament on medium that contains arginine as the sole C- or N-source. We also included the wild-type strain SC5314, the *sky1*Δ mutant and the *SKY1* and *SKY2* complemented strains. On both media the wild type, *sky1*Δ mutant and the *SKY1* complemented strain formed wrinkled colonies which indicate hyphae formation, whereas the *sky2*Δ mutant formed smooth colonies (**Figure 3**). The *SKY2* complemented strain partially rescued the mutant phenotype on both media (**Figure 3**). A similar observation was made in our previous study (Brandt et al., 2022). As discussed there this indicates that one *SKY2* allele is not sufficient to complement the phenotype of the wild-type strain (for detailed data see Supplementary Figure 3 in (Brandt et al., 2022)).

### 3.4 Integrated network analysis links kinase signaling to transcriptional regulation

To investigate the effects of *sky1*Δ and *sky2*Δ knockouts on protein phosphorylation in a network context we integrated previously published mass spectrometry phosphoproteome profiles of *sky1*Δ and *sky2*Δ mutants (Brandt et al., 2022). The experimental conditions (YPD medium; 4 h at 37°C) are concordant with that of our RNA sequencing experiment. The interpretation of phosphorylation events requires knowledge about the kinases potentially responsible for the phosphorylation of a specific protein. Data about such kinase-substrate relationships is sparse in databases, even for established model organisms and especially for *C. albicans* no experimental data seems to be available from databases so far. Therefore, we performed an *in-silico* analysis to predict candidate kinases for each regulated phosphorylation site in the phosphoproteome data using the motif-based approach GPS5 (Group based Prediction system) (Wang et al., 2020) with a high threshold setting to obtain only high confidence predictions (see Material and Methods and **Supplementary Figure 1**). With that we were able to assign a potential kinase to 55.4% of the *sky1*Δ and 33.8% of the *sky2*Δ proteins that were differentially phosphorylated. For *S. cerevisiae* several known kinase-substrate interactions have been experimentally identified and are available in databases. To further complement the set of motif-based predictions for *sky1*Δ and *sky2*Δ we transferred known kinase-substrate interactions of *S. cerevisiae* to *C. albicans* based on protein orthology information (see Materials and Methods). Apart from direct phosphorylation events, protein-protein interactions are an important part of signaling networks. Hence, we collected experimentally validated interactions of *C. albicans* from databases for the set of differentially expressed or phosphorylated proteins, focusing on interactions involving kinases and transcription factors. This yielded 85 interactions between 74 proteins for *sky1*Δ and 51 interactions between 50 proteins for *sky2*Δ.

### 3.5 The transcriptional response of *sky1*Δ and *sky2*Δ is largely driven by a few core transcription factors Ace2, Efg1 and Flo8

**Figure 4:**
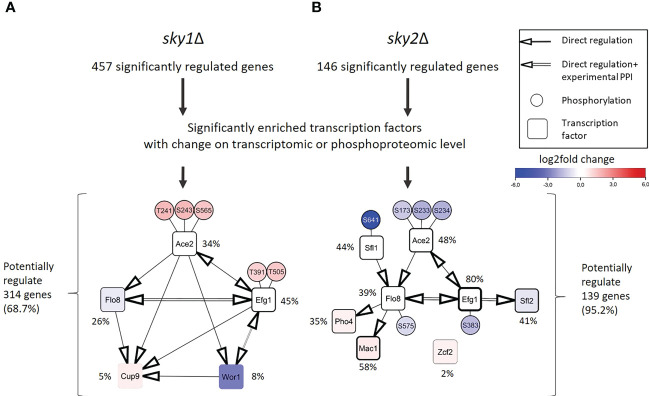
Regulation and protein-protein interaction modules of enriched transcription factors point towards a few core regulators that are differentially phosphorylated or expressed in *sky1*Δ and *sky2*Δ. **(A)** Modules for *sky1*Δ and **(B)** *sky2*Δ enriched transcription factors (rectangles and phosphorylation sites (small circles) that are connected by simple edges representing a known direct regulation of the node, whereas double edges represent a known physical protein-protein interaction (PPI). Circle fill color intensity shows the strength of differential regulation or phosphorylation, where blue color show decreased, red increased and white no significant regulation (wild type vs *sky*Δ; P-value <0.05). Transcription factors are annotated with the relative frequency (percent) of regulated targets of the total significantly regulated genes.

Next, we intended to identify the transcription factors that are driving the observed transcriptional response. Those transcription factors may not be the direct targets of Sky1 and Sky2 and many of the phosphorylated transcription factors may be indirect phosphorylation targets acting downstream in the signaling cascade. Those transcription factors directly bind with the promoters of genes identified as the downstream targets in RNA-seq data. To this end, we performed a transcription factor enrichment analysis for the differentially regulated genes in *sky1*Δ and *sky2*Δ mutants with the PathoYeastract+ (Pathogenic Yeast Search for Transcriptional Regulators And Consensus Tracking) database for *C. albicans* (Monteiro et al., 2020), again focusing on genes with moderate to high expression change (absolute log_2_FC ≥ 0.5 and adjusted P-value < 0.05). In total, we detected a significant enrichment for 45 transcription factors in *sky1*Δ and 67 transcription factors in *sky2*Δ. To identify candidates with a potential impact on transcriptional response in *sky1*Δ and *sky2*Δ, we selected those transcription factors which are either differentially expressed or differentially phosphorylated compared to wild type. This revealed five transcription factors for *sky1*Δ (Ace2, Cup9, Efg1, Flo8, Wor1; **Figure 4A**) and eight transcription factors (Ace2, Efg1, Flo8, Mac1, Pho4, Sfl1, Sfl2, Zcf2) for *sky2*Δ (**Figure 4B**) which together regulate in *sky1*Δ the majority (68.7%; 314/457) and in *sky2*Δ almost all (95.2%; 139/146) genes with moderate to high expression changes. For *sky1*Δ two transcription factors were downregulated and one was upregulated in transcriptomics, whereas two showed an increased phosphorylation (Ace2: T241, S243, S565; Efg1: T391, T505). For *sky2*Δ four transcription factors were transcriptionally regulated with three downregulated and one upregulated while four (Efg1: S383; Ace2: S173, S233, S234; Sfl1: S641; Flo8: S575) showed decreased phosphorylation. This contrasts with *sky1*Δ, where all sites showed an increase in phosphorylation (**Figure 4**). There is evidence in literature that these candidate transcription factors directly or indirectly affect each other in *C. albicans*, either by transcriptional regulation of the corresponding genes (PathoYeastract+) (Monteiro et al., 2020) or by physical protein-protein interaction (IMEx) (Orchard et al., 2012). We compiled a detailed overview of the studies and experimental data supporting these interactions in **Supplementary Table 2** and **3**. With the integration of this information, regulatory modules of these transcription factors can be constructed as depicted in **Figure 4**. For example, Efg1, Flo8, Wor1 and Sfl2 have been described in literature to physically interact (Cao et al., 2006; Noffz et al., 2008; Alkafeef et al., 2018) and have been shown to play a role in hyphal morphogenesis (Stoldt et al., 1997; Cao et al., 2006; Zordan et al., 2007; Nobile et al., 2012; McCall et al., 2018). Here, the transcription factors Ace2, Efg1 and Flo8 are present in both modules as dominating players (based on the number of regulated genes and their location in the network context) For Example, Efg1 directly regulates Ace2, Flo8, Sfl2, Wor1 and Cup9 (Nobile et al., 2012; Hernday et al., 2013), while Flo8 has been shown to directly bind to the promotors of Efg1, Mac1, Cup9 and Pho4 (Znaidi et al., 2013; Fox et al., 2015; Polvi et al., 2019). Based on this data, this set of shared transcription factors has the potential to regulate most of the other enriched transcription factors (Monteiro et al., 2020) (**Figure 4**). Therefore, these transcription factors may be the leading drivers behind the observed transcriptional response.

### 3.6 Ace2, Efg1 and Flo8, show different subsets of regulated genes in *sky1*Δ and *sky2*Δ which might be driven by different phosphorylation, regulation, or modulating interactors

**Figure 5:**
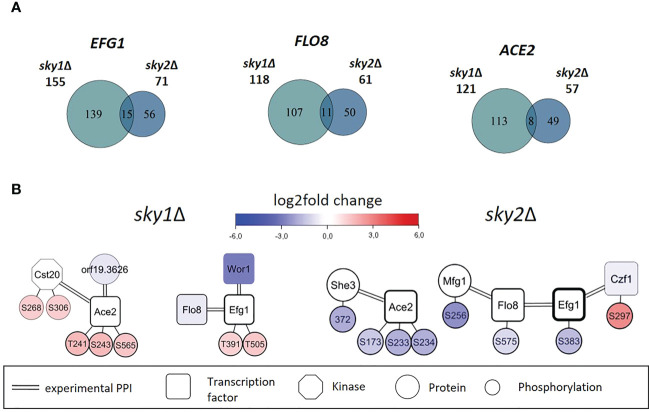
Core set of transcription factors shared by *sky1*Δ and *sky2*Δ show distinct subsets of regulated genes in *sky1*Δ and *sky2*Δ and exhibit different phosphorylation patterns and interactors. **(A)** Venn diagrams for shared transcription factors represents the number of moderately expressed genes that were identified as regulation targets by transcription factor enrichment analysis. **(B)** Transcription factor network of *sky1*Δ (left) and *sky2*Δ (right) with transcription factors (rectangles), potential effector proteins (circles) and phosphorylation sites (small circles) connected by double edges representing a known physical protein-protein interaction. Circle fill color intensity shows the strength of differential regulation or phosphorylation, where blue color shows decreased, red increased and white no significant regulation (wild type vs *sky*Δ, P-value <0.05).

Although these transcription factors together appear to drive large parts of the transcriptional response in both mutant strains the differentially regulated genes targeted by these transcription factors are quite different in *sky1*Δ and *sky2*Δ (**Figure 5A**). This indicates that in the two knockout strains the functionality of these three transcription factors might be modulated by other factors, so that they target different genes. There are several factors by which transcription factors could be influenced; e.g. by post-translational modifications or interacting proteins (Spitz and Furlong, 2012).

The combination of transcriptomic and phosphoproteomic data with literature-derived information on protein-protein and regulatory interactions allows the investigation of individual proteins and phosphorylation events in the context of their interaction environment. With that it is possible to derive novel hypotheses about transcription factors and kinases and their cellular functions. Thus, to identify potential activity regulating factors in *sky1*Δ and *sky2*Δ, we explored the network around these core transcription factors focusing on protein interactors and phosphorylation events (**Figure 5B**). Ace2, Efg1 and Flo8 were differentially phosphorylated in both knockout strains although at different sites and in different directions. Furthermore, the network context provides evidence for several direct protein-protein interactions in *C. albicans* for Ace2 (with Cst20, She3, orf19.3626)(van Wijlick et al., 2016), Flo8 (with Mfg1, Efg1) (Cao et al., 2006; Ryan et al., 2012) and Efg1 (with Flo8, Czf1, Wor1) (Petrovska and Kumamoto, 2012) (see **Figure 5B**; **Supplementary Table 3**). As these interactors were differentially expressed in transcriptomics and/or differentially phosphorylated in the knockout strains phosphoproteomics, they are candidates to act as cofactors to fine-tune the target specificity of the transcription factors.

For example, it has been shown that the binding of Efg1 to its DNA target sequence is influenced by phosphorylation and by interactions with other transcription factors (Lassak et al., 2011). In the network modules (**Figure 5B**) we observe that the Efg1 interaction partner Czf1 (Giusani et al., 2002; Noffz et al., 2008; Petrovska and Kumamoto, 2012; Frazer et al., 2020) is more abundantly phosphorylated in *sky2*Δ and, in addition, Efg1 is differentially phosphorylated in *sky1*Δ and *sky2*Δ. Interestingly, it has been reported that the binding of Czf1 to Efg1 may antagonize it function to change filamentous growth in *C. albicans* (Giusani et al., 2002; Frazer et al., 2020). Here we observe that Czf1 is transcriptionally downregulated in the *sky2*Δ mutant, which might indicate that this antagonistic effect could be weakened in this knockout strain (Petrovska and Kumamoto, 2012). Furthermore, Flo8 is less abundantly phosphorylated in *sky2*Δ at site S575. Albeit this phosphorylation has not been described before, it has been reported, that phosphorylation of the closely located site T589 represses the activity of Flo8 and that dephosphorylation of this site releases that repression (Hollomon et al., 2022). It was reported that Efg1 and Flo8 cooperatively bind to a subset of key targets involved in morphogenesis (Ryan et al., 2012). Furthermore, Mfg1, which is less abundantly phosphorylated in *sky2*Δ but not in *sky1*Δ, has been shown interact directly with Flo8 in *C. albicans* (Ryan et al., 2012).

A central transcription factor in these modules is Ace2 which is not only differentially phosphorylated in both kinase knockouts, but also on different sites and in opposite direction (s*ky1*Δ: T241, S243, S565; *sky2*Δ: S173, S233, S234; **Figure 5B**). These sites are all located in a set of disordered regions between the nuclear export signal and the zinc finger domains (Wakade and Krysan, 2021) with currently unknown effect on the protein. The Ace2 sites S565 and S234 are conserved in *S*. *cerevisiae* (T486; S259) where they have been shown to be phosphorylated by the yeast Cyclin-dependent kinase 1 (CDC28) (Holt et al., 2009), suggesting a conserved functional role for these phosphorylation sites. In *C. albicans* it has been shown that the phosphorylation of Ace2 by Cbk1 plays a role for hyphal morphogenesis and is required for hyphal formation under hypoxic conditions (Wakade et al., 2020). Furthermore, several relevant morphogenetic regulators (Efg1, Flo8, Wor1) in both modules have been shown to be directly regulated by Ace2 (Desai et al., 2015).

Altogether, this indicates, that the transcription factors Ace2, Efg1 and Flo8, are central players in the transcriptional response of both kinases but different regulation of these proteins by phosphorylation or interactions might alter their functionality. In addition all three transcription factors have been reported to be involved in several processes comprising a broader range of cell morphology (Kelly et al., 2004; Cao et al., 2006; Glazier, 2022) in *C. albicans*, supporting the notion that the changes induced by the knockout of *SKY1* and *SKY2* modulate parts of pathways that maintain hyphal development and morphology in different ways.

### 3.7 *Sky1*Δ and *sky2*Δ signaling modules share key network parts but characteristic differences point toward kinase-specific regulation of transcription

**Figure 6:**
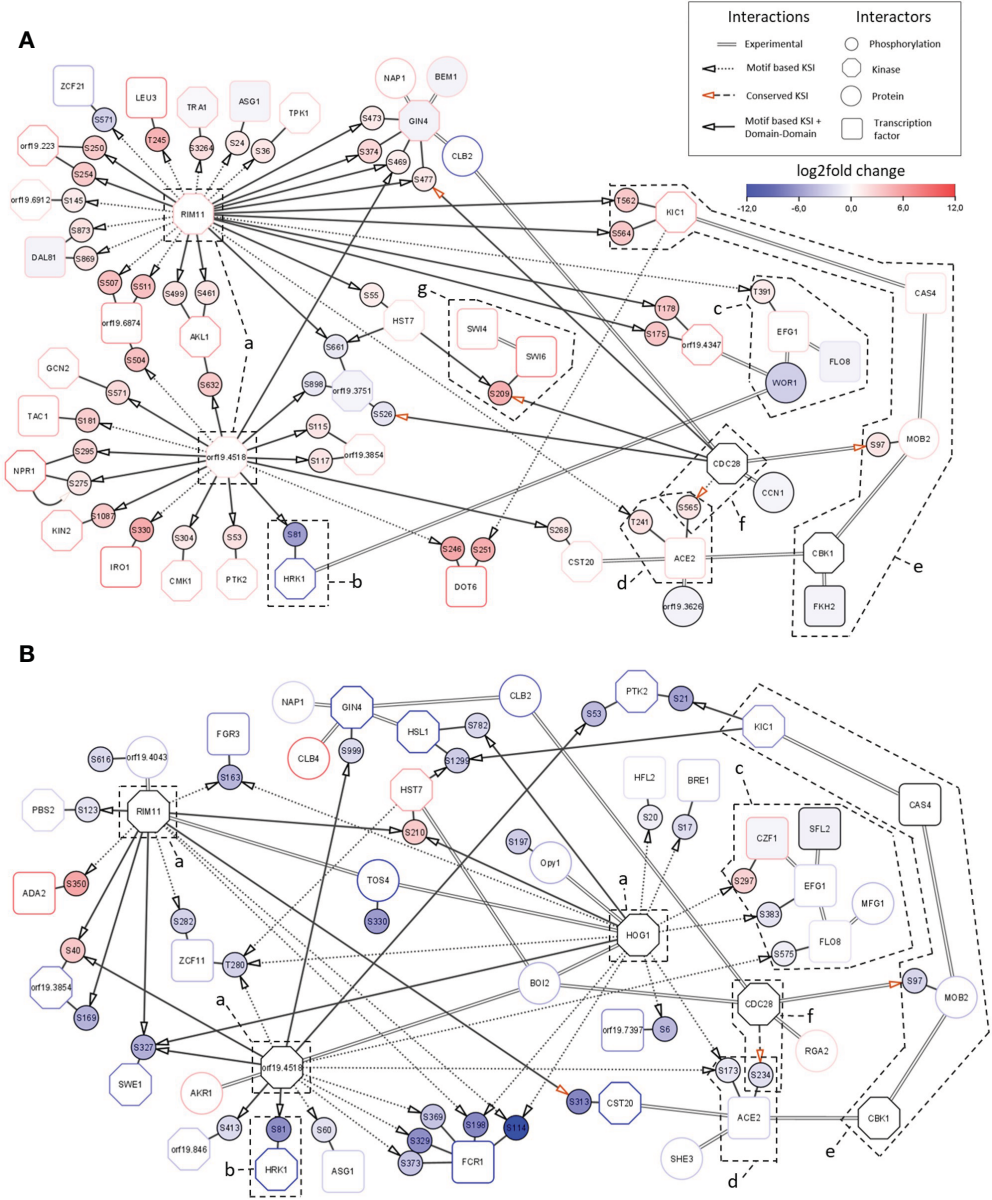
Integrated signaling modules of *sky1*Δ and *sky2*Δ. Network modules of **(A)** s*ky1*Δ and **(B)** *sky2*Δ with kinases (octagons) and transcription factors (rectangles), phosphorylation sites (small circles) and first shell of potential effector proteins (large circles). Border color shows strongest phosphorylation change with significantly decreased (blue) and significantly increased phosphorylation (red; wild type vs *sky*Δ, P-value <0.05). Big node fill color intensity shows the strength of differential transcriptional regulation, where blue color shows down regulation, red upregulation and white no significant regulation (wild type vs *sky*Δ, P-value <0.05). Small node (phosphorylation site) fill color represents the strength of differential phosphorylation, with blue color showing decreased, red increased and white no significant regulation (wild type vs *sky*Δ, P-value <0.05). Kinase-substrate interactions (KSI) are represented by small doted (motif based only) and long doted (conserved only) edges with arrow. KSI supported by domain-domain interactions are shown by a continuous edge while experimentally described interaction are represented by double edges.

The observed transcriptional responses are regulated by transcription factors which are the ultimate effectors of the Sky kinases. Network analysis of phosphoproteome profiles can identify direct and indirect kinase substrates (such as transcription factors). These are not necessarily direct substrates of the Sky kinases but might rather be phosphorylated by other kinases acting downstream in the signaling cascades. To investigate the regulation of the transcriptional responses and elucidate the effects of Sky kinases knockouts, we constructed integrated network modules for both kinase mutants (**Supplementary Figure 1**). The *sky1*Δ signaling module (**Figure 6A**) consists of 51 site-specific direct phosphorylations and 18 experimentally validated protein-protein interactions between 44 proteins. Consistent with the overall direction of phosphorylation change in the *sky1*Δ phosphoproteomic analysis, 89.4% of targeted phosphorylation sites (42/47) display increased phosphorylation. The signaling backbone of this module consists of 22 kinases and 15 transcription factors. Around this core module we found seven additional proteins with experimentally validated interactions in *C. albicans* which could potentially play a modulating role in the signaling cascade (**Figure 6A**). The *sky2*Δ module (**Figure 6B**) contains 16 kinases and 14 transcription factors where most of the 35 phosphorylation sites (31 sites; 88.6%) show a decreased phosphorylation in the mutant (absolute log_2_FC> 1 and adjusted P-Value < 0.05). Both networks are dominated by a few kinases (orf19.4518, Rim11, Hog1; **Figures 6A, B-a**), with orf19.4518 and Rim11 shared by *sky1*Δ and *sky2*Δ, and Hog1 present only in the *sky2*Δ module. Except for Hog1, not much is known for these kinases, but all three have been associated with cellular responses to osmotic stress and cell morphology in *C. albicans.* For example, Hog1 has been described to be involved in osmotic, heavy metal, and core stress response and has a role in regulation of response to stress in *C. albicans* (Smith et al., 2004; Enjalbert et al., 2006; Alonso-Monge et al., 2009). In addition, Rim11 orthologs in *S. cerevisiae* are known to be involved in oxidative stress resistance, resistance to chemicals and filamentous growth (Frohlich et al., 2015; Helsen et al., 2020). Notably, one of the targets of the central kinase orf19.4518 is the site S81 of Hrk1 (**Figures 6A, B-b**) which shows a particularly high decrease (64 times) of phosphorylation in both *Sky* knockout strains. Hrk1 is a putative serine/threonine kinase with a predicted role in cellular ion homeostasis which has previously been described to have the same knockout phenotype as *sky1*Δ (Brandt et al., 2022). Based on this, it was suggested that Hrk1 is either directly phosphorylated by Sky1 or is an indirect target acting in the same pathway. Here, the proposed integrated modules indicate that Hrk1 is rather an indirect target of Sky1 and Sky2 which might instead be directly phosphorylated by the kinase orf19.4518.

Four transcription factors which have been detected in the transcription factor enrichment analysis (**Figures 4**, **5**) as candidate drivers of the *sky1*Δ and *sky2*Δ transcriptional response are also present in the signaling network module. There, Efg1, Flo8 and Wor1 form an interaction complex (**Figures 6A, B-c**, **Supplementary Table 2**) (Cao et al., 2006; Noffz et al., 2008; Alkafeef et al., 2018). In addition Ace2 is integrated in the modules (**Figures 6A, B-d**) as part of the Regulation of Ace2 and Morphogenesis (RAM) pathway (Wakade and Krysan, 2021). Interestingly, these core transcription factors seem to be targeted by orf19.4518, Rim11 and Hog1 in different ways with phosphorylation effects in mostly opposite directions in *sky1*Δ and *sky2*Δ. Based on motif-based kinase substrate analyses, only Efg1 is phosphorylated by Rim11 in the *sky1*Δ module, whereas three transcription factors (Czf1: S297; Efg1: S383; Flo8: S575) are targeted by the kinases Hog1 and orf19.4518 in the in sky2Δ module. This indicates a differential adjustment of Rim11, orf19.4518 and Hog1 in *sky1*Δ compared to *sky2*Δ potentially leading to distinct downstream effects on the transcriptional responses.

**Figure 7:**
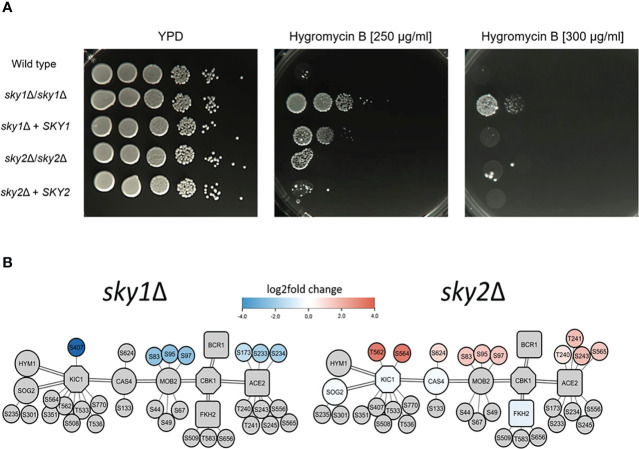
Deletion of *SKY1* confers resistance to Hygromycin B and both Sky knockouts strains show distinct changes of the RAM-Pathway in transcriptomics and phosphoproteomics. **(A)** YPD overnight cultures of the strains were adjusted to an optical density (OD_600_) of 1.0 and 5 µl of serial 10-fold dilutions and were spotted on YPD agar plates without or with 250 µg/ml or 300 µg/ml Hygromycin B and incubated for 3 days at 37°C. The experiment was performed in duplicates and representative images are shown. **(B)** Overview of the components of the RAM pathway of *sky1*Δ (left) and *sky2*Δ (right) with kinases (octagons) and transcription factors (rectangles), phosphorylation sites (small circles) and first shell of potential effector proteins (large circles). Big node fill color intensity shows the strength of differential transcriptional regulation, where blue color shows down regulation, red upregulation and grey no significant regulation (wild type vs *sky*Δ, P-value <0.05). Small node fill color represents the strength of differential phosphorylation, with blue color showing decreased, red increased and grey no significant regulation (wild type vs *sky*Δ, P-value <0.05).

For Ace2, the network modules depict the phosphorylation of the sites S565 (log_2_FC = 1.5 and adjusted P-value < 0.05) in *sky1*Δ and S234 (log_2_FC = -1.9 and adjusted P-value < 0.05) in *sky2*Δ which are both conserved in *S. cerevisiae* and are targeted there by the kinase Cdc28 (**Figures 6A, B-f**) (Koivomagi et al., 2011; Faustova et al., 2021). In addition to Ace2, our signaling modules integrate further interactors of the RAM pathway (Hym1, Sog2, Kic1, Cas4, Mob2, Cbk1, Fkh2, Bcr1; **Figures 6A, B-e**, **7B**) (Wakade and Krysan, 2021). Several interactors of this pathway are present in both modules but with different changes in *sky1*Δ and *sky2*Δ. Some components (Kic1, Fkh2, Cas4) are transcriptionally downregulated in *sky1*Δ but none of the genes are transcriptionally regulated in *sky2*Δ. On the protein level a decreased phosphorylation of Kic1, Mob2 can be observed in *sky1*Δ (compared to the wild type), in contrast to an increased phosphorylation in *sky2*Δ (**Figure 7B**). Another phosphorylation target of Cdc28 in both modules is Mob2 site S97 (supported by motif-based kinase substrate analysis and domain domain-interaction). Phosphorylation of this site has previously been shown to be crucial for normal hyphal development on hyphae-inducing conditions in *C. albicans* (Gutierrez-Escribano et al., 2011). However, in contrast to the *sky2*Δ module, the *sky1*Δ module also contains three other potential substrates targeted by Cdc28 namely Swi6, Gin4 and orf19.3751 (**Figure 6**; supported by motif-based kinase substrate analysis and domain domain-interaction). Swi6, together with Swi4, is a core component of the conserved (*S. cerevisiae*) (Bahler, 2005) cell cycle box-binding factor (SBF) complex (Hussein et al., 2011) (**Figures 6A–G**), which plays an important role in cell proliferation, G_1_/S progression and may be linked to aspects of hyphal development. Furthermore, the phosphorylation of Swi6 site S209 by Cdc28 is also conserved in *S. cerevisiae*. Furthermore, in addition to Swi6, Swi4 is differentially phosphorylated only in *sky1*Δ (log_2_FC = 2.7 and adjusted P-value < 0.05) but not in *sky2*Δ, suggesting that *sky1*Δ and *sky2*Δ differentially fine-tune Cdc28 kinase activity to regulate the RAM pathway and SBF complex. Since Swi4 and Swi6, similar to Ace2, influence morphogenesis including hyphae differentiation in *C. albicans* (Hussein et al., 2011) this could explain the opposing roles of Sky1 and Sky2 in these cell processes through direct or indirect regulation of Cdc28 activity. In line with this, an analysis of the network modules in the context of knockout phenotypes (based on literature and databases)(Skrzypek et al., 2017) revealed that a large number of genes in the modules of *sky1*Δ and *sky2*Δ are associated with cell morphological functional categories. For example, 65.9% (29/44) in the *sky1*Δ and 40.0% (30/75) of the proteins in the *sky2*Δ module are connected to filamentous growth. Additionally, 72% of the *sky1*Δ proteins are associated with resistance to chemicals whereas only 30% of the *sky2*Δ proteins were associated with this knockout phenotype.

To test whether *sky1*Δ and *sky2*Δ differentially influence the function of the RAM pathway, we tested the susceptibility of both mutants for Hygromycin B. This aminoglycoside antibiotic inhibits the protein synthesis and translation of both prokaryotic and eukaryotic cells and it has been shown that knockout of genes of the RAM pathway (*CBK1*, *HYM1*, *KIC1*, *MOB2*, *PAG1*, *SOG2*) induces hypersensitivity (Song et al., 2008). The *sky1*Δ mutant was less susceptible to Hygromycin B compared to the wild type and the *sky2*Δ mutant (**Figure 7A**). As the *sky1*Δ signaling module demonstrates that parts of the RAM pathway are differentially phosphorylated and regulated in the *sky1*Δ mutant (**Figure 7B**), this might explain the observed phenotype on Hygromycin B.

## 4 Conclusion

Here we investigated the different functions of the *C. albicans* Sky1 and Sky2 kinases regarding transcriptional regulation, kinase signaling and phenotype within an integrated network context. Our analyses revealed, that *sky1*Δ and *sky2*Δ show distinct transcriptional responses and share only a small number of regulated genes. These seems to be regulated by a common set of core transcription factors that have the potential to regulate large but Sky kinase-specific subsets of target genes. However, these core transcription factors and their adjacent protein interactors show mostly opposite or different phosphorylation and expression status, indicating potential mechanisms to fine tune the regulation of their target genes. Additionally, transcriptomic enrichment in *sky1*Δ reveals functional categories similar to the known role of Sky1 orthologs in *S. cerevisiae* namely pre-mRNA splicing, mRNA export from the nucleus and mitochondrial respiratory chain association, further supportingthe proposed conserved role of this kinase in *C. albicans*. In contrast, Sky2 seems to have a different role as suggested by enrichment of genes in the cell periphery and glutamine family amino acid metabolic process, supporting the prior observed role in nitrogen compound metabolic processes. In particular, *sky2*Δ shows an upregulation of several proteins of the arginine biosynthesis pathways while a large part of the arginine catabolic pathway is downregulated. Experimental validation of these observations revealed that arginine-induced hyphae formation is impaired in the *sky2*Δ mutant. Furthermore, transcription factor enrichment and integrated analysis of kinase signaling networks point towards several key components in the *SKY1* and *SKY2* knockout response which are connected to fungal morphology, resistance to chemicals and stress for both *C. albicans* Sky kinases. Further experimental phenotyping delivered evidence that *sky1*Δ results in higher resistance to Hygromycin B confirming these observations. Altogether, our results consolidate the hypotheses that in *C. albicans* the Sky1 protein kinase is functionally similar to Sky1 in *S. cerevisiae*, whereas *C. albicans* Sky2 seems to have distinct functions regarding gene expression and signaling. These might be controlled by transcriptional and posttranslational regulation of key transcription factors and effector proteins. Our data and network offer a systems biological perspective of candida phosphorylation and thus can help to generate novel hypotheses and obtain deeper insights into mechanisms of complex signaling cascades and transcriptional regulation.

## Data availability statement

The data presented in the study are deposited in the Sequence Read Archive (SRA) at NCBI (https://www.ncbi.nlm.nih.gov/sra) under the accession number PRJNA941841.

## Author contributions

CL, MD, TM analyzed the data. PB performed the wet lab experiments. CL, PB designed the figures. MD, TD, TM, SV discussed the results. MD, SV, TD supervised the project. CL, MD, PB wrote the manuscript in consultation with SV, TD, TM. All authors contributed to the article and approved the submitted version.
