# Supplementary material for: Integrated analysis of SR-like protein kinases Sky1 and Sky2 links signaling networks with transcriptional regulation in Candida albicans
Source: Front Cell Infect Microbiol. 2023 Apr 3;13:1108235. doi: 10.3389/fcimb.2023.1108235 (PMC10111165; doi:10.3389/fcimb.2023.1108235)
Supplement: Supplementary file 1 [file DataSheet_1.pdf]

Supplementary Material

1 Supplementary Figures and Tables

1.1 Supplementary Figures

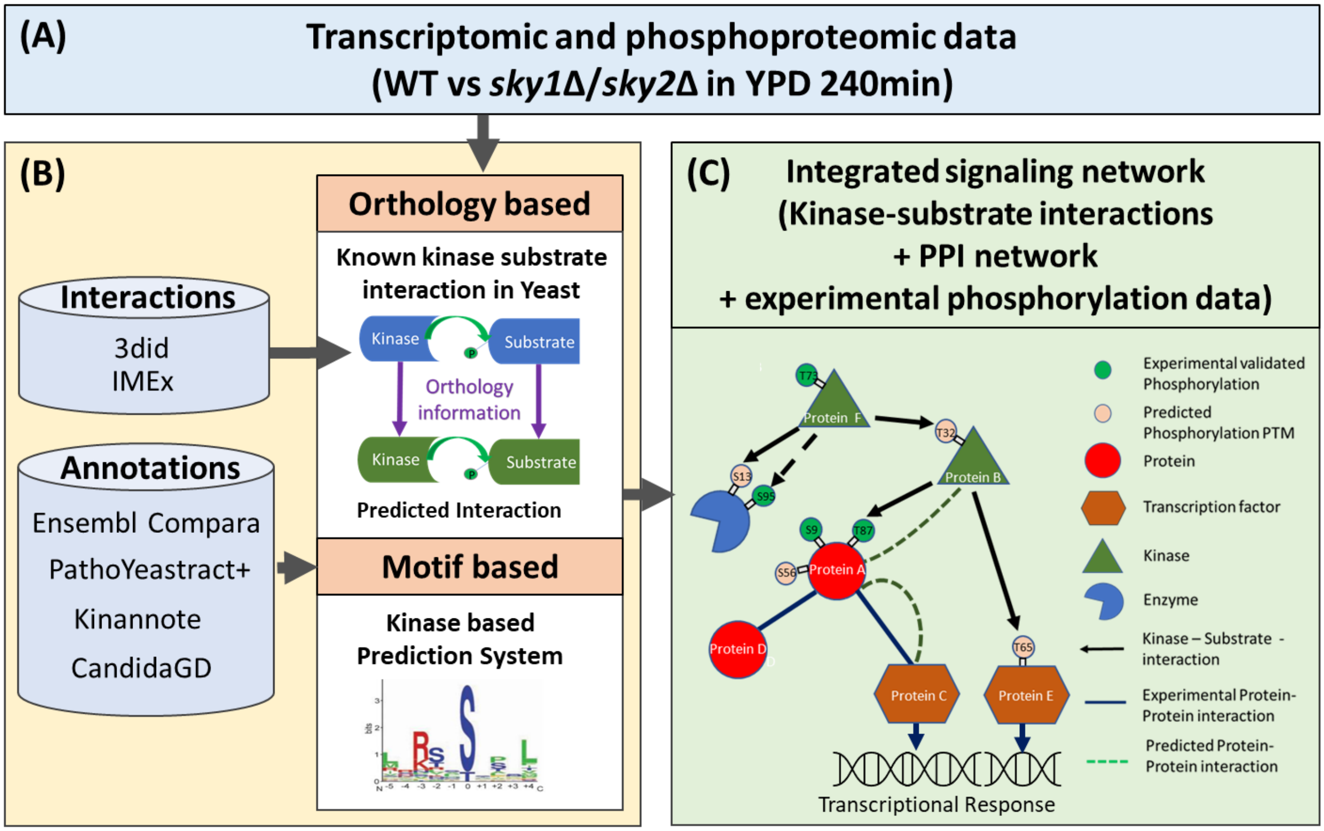

Supplementary Figure 1: Concept of the integrated signaling module construction. (A) Quantitative differential gene expression and phosphorylation analysis of *sky1*Δ and *sky2*Δ versus WT in YPD after 4 hours. **(B)** For differentially expressed and phosphorylated genes and proteins, site-specific kinase-substrate interactions predictions were performed by interolog transfer as well as sequence motif-based analysis and complemented by the collection of experimentally known protein-protein interactions from literature. **(C)** Construction of a *sky1*Δ and *sky2*Δ integrated signaling modules based on predicted und experimentally validated interactions and omics data

2 Supplementary Tables

Supplementary Table 1: *C. albicans* strains used in this study

Strain	Genotype	Parental strain	Reference
**SC5314**	wild type	–	(Gillum et al., 1984)
***sky1*Δ A**	*sky1*Δ::*FRT*/*sky1*Δ::*FRT*	SC5314	(Ramirez-Zavala et al., 2022)
***sky2*Δ A**	*sky2*Δ::*FRT*/*sky2*Δ::*FRT*	SC5314	(Ramirez-Zavala et al., 2017)
***sky1*Δ + *SKY1* A**	*sky1*Δ::*FRT*/*SKY1*-*FRT*	*sky1*Δ A	(Brandt et al., 2022)
***sky2*Δ + *SKY2* A**	*sky2*Δ::*FRT*/*SKY2*-*FRT*	*sky2*Δ A	(Brandt et al., 2022)

Supplementary Table 2: Detailed information for direct protein-protein interactions (Figure 4,5) based on IMEx (Orchard et al., 2012)

Interactor A	Interactor B	Experimental system	References	Species
Ace2	Cst20	Two-hybrid, Affinity Capture-Western	(van Wijlick et al., 2016)	*C. albicans*
Ace2	orf19.3626	Two-hybrid	(van Wijlick et al., 2016)	*C. albicans*
Ace2	She3	Two-hybrid	(van Wijlick et al., 2016)	*C. albicans*
Efg1	Czf1	Two-hybrid	(Giusani et al., 2002; Noffz et al., 2008; Petrovska and Kumamoto, 2012; Frazer et al., 2020)	*C. albicans*
Efg1	Flo8	Two-hybrid, Affinity Capture-Western	(Cao et al., 2006; Noffz et al., 2008)	*C. albicans*
Efg1	Sfl2	Affinity Capture-Western	(Znaidi et al., 2013)	*C. albicans*
Efg1	Wor1	Affinity Capture-MS	(Alkafeef et al., 2018)	*C. albicans*
Flo8	Efg1	Two-hybrid, Affinity Capture-Western	(Cao et al., 2006; Noffz et al., 2008)	*C. albicans*
Flo8	Mfg1	Affinity Capture-MS	(Ryan et al., 2012)	*C. albicans*

Supplementary Table 3: Detailed information for direct regulatory interactions (Figure 4) based on PathoYeastract+ (Monteiro et al., 2020)

Transcription factor	Target gene	Regulation type	Experimental system	References	Species
Ace2	Cup9	Direct	Chip-on-chip	(Desai et al., 2015)	*C. albicans*
Ace2	Efg1	Direct	Chip-on-chip	(Desai et al., 2015)	*C. albicans*
Ace2	Flo8	Direct	Chip-on-chip	(Desai et al., 2015)	*C. albicans*
Ace2	Wor1	Direct	Chip-on-chip	(Desai et al., 2015)	*C. albicans*
Efg1	Ace2	Direct	Chip-on-chip	(Saputo et al., 2014)	*C. albicans*
Efg1	Cup9	Direct	Chip-on-chip	(Lassak et al., 2011; Nobile et al., 2012; Desai et al., 2015)	*C. albicans*
Efg1	Flo8	Direct	Chip-on-chip	(Nobile et al., 2012)	*C. albicans*
Efg1	Sfl2	Direct	Chip-on-chip	(Nobile et al., 2012)	*C. albicans*
Efg1	Wor1	Direct	Chip-on-chip	(Lassak et al., 2011; Nobile et al., 2012; Hernday et al., 2013)	*C. albicans*
Flo8	Cup9	Direct	Chip-on-chip	(Polvi et al., 2019)	*C. albicans*
Flo8	Efg1	Direct	ChIP	(Fox et al., 2015)	*C. albicans*
Flo8	Mac1	Direct	Chip-on-chip	(Polvi et al., 2019)	*C. albicans*
Flo8	Pho4	Direct	Chip-on-chip	(Polvi et al., 2019)	*C. albicans*
Sfl1	Flo8	Direct	ChIP	(Znaidi et al., 2013)	*C. albicans*
Wor1	Cup9	Direct	ChIP	(Zordan et al., 2007; Hernday et al., 2013)	*C. albicans*
Wor1	Efg1	Direct	RT-PCR - wt vs mutant promoter sequence, ChIP, Tandem affinity HBH purification coupled with mass spectroscopy	(Zordan et al., 2006; Zordan et al., 2007; Hernday et al., 2013; Alkafeef et al., 2018)	*C. albicans*

References

Alkafeef, S.S., Yu, C., Huang, L., and Liu, H. (2018). Wor1 establishes opaque cell fate through inhibition of the general co-repressor Tup1 in Candida albicans. *PLoS Genet* 14(1)**,** e1007176. doi: 10.1371/journal.pgen.1007176.

Brandt, P., Gerwien, F., Wagner, L., Kruger, T., Ramirez-Zavala, B., Mirhakkak, M.H., et al. (2022). Candida albicans SR-Like Protein Kinases Regulate Different Cellular Processes: Sky1 Is Involved in Control of Ion Homeostasis, While Sky2 Is Important for Dipeptide Utilization. *Front Cell Infect Microbiol* 12**,** 850531. doi: 10.3389/fcimb.2022.850531.

Cao, F., Lane, S., Raniga, P.P., Lu, Y., Zhou, Z., Ramon, K., et al. (2006). The Flo8 transcription factor is essential for hyphal development and virulence in Candida albicans. *Mol Biol Cell* 17(1)**,** 295-307. doi: 10.1091/mbc.e05-06-0502.

Desai, P.R., van Wijlick, L., Kurtz, D., Juchimiuk, M., and Ernst, J.F. (2015). Hypoxia and Temperature Regulated Morphogenesis in Candida albicans. *PLoS Genet* 11(8)**,** e1005447. doi: 10.1371/journal.pgen.1005447.

Fox, E.P., Bui, C.K., Nett, J.E., Hartooni, N., Mui, M.C., Andes, D.R., et al. (2015). An expanded regulatory network temporally controls Candida albicans biofilm formation. *Mol Microbiol* 96(6)**,** 1226-1239. doi: 10.1111/mmi.13002.

Frazer, C., Staples, M.I., Kim, Y., Hirakawa, M., Dowell, M.A., Johnson, N.V., et al. (2020). Epigenetic cell fate in Candida albicans is controlled by transcription factor condensates acting at super-enhancer-like elements. *Nat Microbiol* 5(11)**,** 1374-1389. doi: 10.1038/s41564-020-0760-7.

Gillum, A.M., Tsay, E.Y., and Kirsch, D.R. (1984). Isolation of the Candida albicans gene for orotidine-5'-phosphate decarboxylase by complementation of S. cerevisiae ura3 and E. coli pyrF mutations. *Mol Gen Genet* 198(2)**,** 179-182. doi: 10.1007/BF00328721.

Giusani, A.D., Vinces, M., and Kumamoto, C.A. (2002). Invasive filamentous growth of Candida albicans is promoted by Czf1p-dependent relief of Efg1p-mediated repression. *Genetics* 160(4)**,** 1749-1753. doi: 10.1093/genetics/160.4.1749.

Hernday, A.D., Lohse, M.B., Fordyce, P.M., Nobile, C.J., DeRisi, J.L., and Johnson, A.D. (2013). Structure of the transcriptional network controlling white-opaque switching in Candida albicans. *Mol Microbiol* 90(1)**,** 22-35. doi: 10.1111/mmi.12329.

Lassak, T., Schneider, E., Bussmann, M., Kurtz, D., Manak, J.R., Srikantha, T., et al. (2011). Target specificity of the Candida albicans Efg1 regulator. *Mol Microbiol* 82(3)**,** 602-618. doi: 10.1111/j.1365-2958.2011.07837.x.

Monteiro, P.T., Oliveira, J., Pais, P., Antunes, M., Palma, M., Cavalheiro, M., et al. (2020). YEASTRACT+: a portal for cross-species comparative genomics of transcription regulation in yeasts. *Nucleic Acids Res* 48(D1)**,** D642-D649. doi: 10.1093/nar/gkz859.

Nobile, C.J., Fox, E.P., Nett, J.E., Sorrells, T.R., Mitrovich, Q.M., Hernday, A.D., et al. (2012). A recently evolved transcriptional network controls biofilm development in Candida albicans. *Cell* 148(1-2)**,** 126-138. doi: 10.1016/j.cell.2011.10.048.

Noffz, C.S., Liedschulte, V., Lengeler, K., and Ernst, J.F. (2008). Functional mapping of the Candida albicans Efg1 regulator. *Eukaryot Cell* 7(5)**,** 881-893. doi: 10.1128/EC.00033-08.

Orchard, S., Kerrien, S., Abbani, S., Aranda, B., Bhate, J., Bidwell, S., et al. (2012). Protein interaction data curation: the International Molecular Exchange (IMEx) consortium. *Nat Methods* 9(4)**,** 345-350. doi: 10.1038/nmeth.1931.

Petrovska, I., and Kumamoto, C.A. (2012). Functional importance of the DNA binding activity of Candida albicans Czf1p. *PLoS One* 7(6)**,** e39624. doi: 10.1371/journal.pone.0039624.

Polvi, E.J., Veri, A.O., Liu, Z., Hossain, S., Hyde, S., Kim, S.H., et al. (2019). Functional divergence of a global regulatory complex governing fungal filamentation. *PLoS Genet* 15(1)**,** e1007901. doi: 10.1371/journal.pgen.1007901.

Ramirez-Zavala, B., Kruger, I., Dunker, C., Jacobsen, I.D., and Morschhauser, J. (2022). The protein kinase Ire1 has a Hac1-independent essential role in iron uptake and virulence of Candida albicans. *PLoS Pathog* 18(2)**,** e1010283. doi: 10.1371/journal.ppat.1010283.

Ramirez-Zavala, B., Mottola, A., Haubenreisser, J., Schneider, S., Allert, S., Brunke, S., et al. (2017). The Snf1-activating kinase Sak1 is a key regulator of metabolic adaptation and in vivo fitness of Candida albicans. *Mol Microbiol* 104(6)**,** 989-1007. doi: 10.1111/mmi.13674.

Ryan, O., Shapiro, R.S., Kurat, C.F., Mayhew, D., Baryshnikova, A., Chin, B., et al. (2012). Global gene deletion analysis exploring yeast filamentous growth. *Science* 337(6100)**,** 1353-1356. doi: 10.1126/science.1224339.

Saputo, S., Kumar, A., and Krysan, D.J. (2014). Efg1 directly regulates ACE2 expression to mediate cross talk between the cAMP/PKA and RAM pathways during Candida albicans morphogenesis. *Eukaryot Cell* 13(9)**,** 1169-1180. doi: 10.1128/EC.00148-14.

van Wijlick, L., Swidergall, M., Brandt, P., and Ernst, J.F. (2016). Candida albicans responds to glycostructure damage by Ace2-mediated feedback regulation of Cek1 signaling. *Mol Microbiol* 102(5)**,** 827-849. doi: 10.1111/mmi.13494.

Znaidi, S., Nesseir, A., Chauvel, M., Rossignol, T., and d'Enfert, C. (2013). A comprehensive functional portrait of two heat shock factor-type transcriptional regulators involved in Candida albicans morphogenesis and virulence. *PLoS Pathog* 9(8)**,** e1003519. doi: 10.1371/journal.ppat.1003519.

Zordan, R.E., Galgoczy, D.J., and Johnson, A.D. (2006). Epigenetic properties of white-opaque switching in Candida albicans are based on a self-sustaining transcriptional feedback loop. *Proc Natl Acad Sci U S A* 103(34)**,** 12807-12812. doi: 10.1073/pnas.0605138103.

Zordan, R.E., Miller, M.G., Galgoczy, D.J., Tuch, B.B., and Johnson, A.D. (2007). Interlocking transcriptional feedback loops control white-opaque switching in Candida albicans. *PLoS Biol* 5(10)**,** e256. doi: 10.1371/journal.pbio.0050256.
